# Variation and gaps in online pharmacy regulation: a comparative review of regulations across 12 countries

**DOI:** 10.1136/bmjph-2025-004309

**Published:** 2026-09-18

**Authors:** Gautam Satheesh, Krishna Nandakumar, Benjamin Palafox, Jaison Joseph, Sushree Nibedita Panda, Devaki Nambiar, Abdul Salam, Sammy Masibo, Francis P N Wafula, Maria-Belen Tarrafeta-Sayas, Raffaella Ravinetto, Catherine Goodman

**Affiliations:** 1 The George Institute for Global Health India, Hyderabad, India; 2 University of Sydney Faculty of Medicine and Health, Sydney, New South Wales, Australia; 3 Department of Global Health and Development, London School of Hygiene and Tropical Medicine, London, UK; 4 The George Institute for Global Health India, New Delhi, India; 5 Faculty of Medicine, UNSW Sydney, Sydney, New South Wales, Australia; 6 Prasanna School of Public Health, Manipal Academy of Higher Education, Manipal, India; 7 The George Institute for Global Health, UNSW Sydney, Sydney, New South Wales, Australia; 8 Prism Centre, Strathmore University Business School, Nairobi, Kenya; 9 Department of Public Health, Institute of Tropical Medicine, Antwerp, Belgium; 10 School of Public Health, University of the Western Cape, Cape Town, South Africa

**Keywords:** Health Services Accessibility, Public Health, legislation and jurisprudence, Digital Health

## Abstract

**Objectives:**

E-pharmacies carry enormous potential to improve access to medicines but also introduce significant regulatory concerns. This research maps the regulatory approaches governing e-pharmacies across 12 countries and identifies gaps in their scope and comprehensiveness.

**Design:**

Comparative review of regulatory documents.

**Data sources:**

Publicly accessible documents from governments, regulatory authorities and relevant national, subnational and supranational sources.

**Eligibility criteria:**

Countries (Brazil, Germany, Ghana, Indonesia, Ireland, Japan, Kenya, Singapore, South Africa, UAE, UK and USA) were purposively selected to capture geographic and regulatory diversity.

**Data extraction and synthesis:**

We extracted e-pharmacy-related regulatory text using a piloted tool, categorising identified requirements across 10 domains: licensure, accreditation, medicine sale, website content, data privacy, quality, operational oversight, advertising, penalties and other technological aspects. We also developed a 15-item subset of basic requirements and assessed regulatory comprehensiveness.

**Results:**

From 41 documents, we identified 43 regulatory requirements, with substantial country and regional variation in whether each was mandated, permitted, prohibited or unaddressed. Online sale of prescription medicines was explicitly permitted in 8 of 12 countries, but prohibited in Ireland, Japan and UAE. Most countries (n=9) required e-pharmacies to be linked to a brick-and-mortar pharmacy. Four countries prohibited online sale of narcotics. Approaches for verifying website authenticity included displaying licence numbers (n=5) and using a ‘common logo’ (n=5). Fewer regulations addressed newer technological developments, including mobile apps (n=2) and social media (n=1). Kenya and the UK met all requirements on our 15-item checklist, indicating higher comprehensiveness.

**Conclusion:**

We identified substantial variation in how e-pharmacy regulations addressed the 10 regulatory domains, particularly licensure, medicine sale and website authenticity. Gaps in regulatory coverage were most evident for accreditation, penalties and integration with broader digital health systems. This review offers a practical checklist of basic regulatory requirements to help policymakers strengthen e-pharmacy governance and ensure safe access to medicines online.

**WHAT IS ALREADY KNOWN ON THIS TOPIC**

**WHAT THIS STUDY ADDS**

- Our comparative review of national, subnational and supranational e-pharmacy regulatory documents across 12 countries identified 43 regulatory requirements spanning 10 key domains and informed a 15-item checklist of basic requirements to assess comprehensiveness.
- We identified substantial variation in how e-pharmacy regulations addressed the 10 domains, particularly licensure, medicine sale and website authenticity, with key gaps in accreditation, penalties, newer technological aspects and integration with broader digital health systems.

**HOW THIS STUDY MIGHT AFFECT RESEARCH, PRACTICE OR POLICY**

- Our findings provide a valuable resource for regulatory authorities seeking to develop and/or strengthen their regulations, by consolidating information on the regulatory models currently in place across a wide range of countries and settings.
- Policy priorities include clear licensing and verification processes, specific rules for online sale of prescription and controlled medicines, and integration of e-pharmacy oversight within broader data protection and digital systems, adapted to different governance contexts (national, supranational and subnational) and supported by adequate regulatory capacity.

## Background

 The global online pharmacy market is expanding rapidly—valued at US$125 billion in 2026 and projected to reach US$310 billion by 2031—with emerging markets in low- and middle-income countries (LMICs) driving much of this surge.^1^ This pattern aligns with the growing use of digital technologies across health systems, including telemedicine and electronic health records. Online pharmacies (referred to henceforth as e-pharmacies) have the potential to expand access to medicines across populations, especially the elderly, disabled and those living in rural areas or ‘pharmacy deserts’.^2^

However, this rapid expansion has largely preceded regulatory development, particularly in LMICs, raising concerns about public health risks, including sale of prescription-only medicines (POMs) without prescription, inadequate provision of information to clients, challenges to data privacy and consumer fraud.^3,4^ In a 2021 survey, about half of 79 surveyed countries (41 LMICs and 38 high-income countries (HICs)) had no regulations specific to e-pharmacies.^5^ A more recent study found a similar situation, reporting that only 5 of 15 (mainly LMICs) countries studied had advanced regulations.^6^ While many HICs and middle-income countries (MICs) have developed regulations aimed at mitigating these risks, regulatory challenges persist worldwide, including low regulatory capacity, cross-border regulatory enforcement challenges and concerns over risk of over-regulation.^3^ Ensuring regulatory compliance remains an uphill battle for regulators.^7^ Even countries thought to have mature e-pharmacy regulations face challenges, including the growing threat of illegal or ‘rogue’ players resembling legitimate providers and operating without licensure, selling falsified, unlicensed or substandard products and failing to comply with medicine/patient safety regulations.^8–10^ Meanwhile, many LMICs continue to struggle to ensure compliance among e-pharmacies and brick-and-mortar pharmacies, with both sectors often operating without qualified personnel, selling POMs without prescription and failing to provide adequate information to patients.^11,12^

While recognising that challenges in regulating e-pharmacy exist worldwide, countries with relatively advanced e-pharmacy regulations may offer lessons for those still in the process of developing their regulatory frameworks. Although these insights need to be tailored to fit the regulatory, legal and market environment in each country, they may aid in understanding the range of potential regulatory approaches and identifying best practices.

Previous reports comparing e-pharmacy regulations have highlighted substantial variation across settings, including within and across geographical regions and income groups (online supplemental table 1). Early reviews explored medicine shipping regulations and guidance for acquirers, that is, financial institutions, to onboard e-pharmacy services,^13,14^ while others summarised accreditation systems and regulatory approaches in selected countries in Asia,^15^ Africa,^3^ Latin America^16^ and the Gulf.^17^ A 2025 viewpoint examined ethical guidance for pharmacists working in e-pharmacies but was limited to the European Union (EU) and focused specifically on professional ethics.^18^ In addition, two telemedicine reviews in Asia-Pacific countries provide limited information on e-pharmacy.^19,20^ Many of the above reviews are outdated, given the rapidly changing regulatory landscape, and none covered all global regions or examined all aspects of e-pharmacy regulations in depth.

To address these gaps, we aimed to compare regulatory approaches governing e-pharmacies across 12 countries with enacted e-pharmacy-specific regulations, and to identify gaps in the scope and comprehensiveness of regulation in order to inform ongoing regulatory development in these and other settings.

## Methods

We conducted a comparative review of regulations relating directly to e-pharmacies at the supranational, national and subnational levels, including both mandatory measures and voluntary requirements.

### Country selection

We purposively selected 12 countries to capture a broad range of regulatory models (table 1). Countries were eligible if they had enacted e-pharmacy-specific regulations as of January 2025, and had publicly available, e-pharmacy-specific regulatory or legal documents. The choice of the number of countries included was pragmatic, balancing the need to ensure feasibility for in-depth document review, with the aim to cover all main regulatory approaches (eg, whether online sale of prescription-only medicines is permitted; whether regulation is devolved to subnational level). Selection was informed by the existing literature (online supplemental table 1) and consultation with international experts on e-pharmacy, including from the Alliance for Safe Online Pharmacy and the USA’s National Association of Boards of Pharmacy (NABP). We also aimed to achieve broad geographical coverage, including countries in six of the seven World Bank regions^21^ (no countries in the South Asia region had enacted e-pharmacy-specific regulations). (table 1) This included seven HICs, three upper-MICs and two lower-MICs.

**Table 1:** Selected countries included in assessment

World Bank region	Countries	Income classification
Latin America and the Caribbean	Brazil	U-MIC
North America	USA (plus three focus states)	HIC
Europe and Central Asia	UK Germany Ireland	HIC HIC HIC
East Asia and Pacific	Indonesia Singapore Japan	U-MIC HIC HIC
Sub-Saharan Africa	Ghana South Africa Kenya	L-MIC U-MIC L-MIC
Middle East and North Africa	United Arab Emirates (plus Dubai)	HIC
South Asia*	–	–

* None of the eight countries in this region had e-pharmacy regulation. Some had consensus documents or reports, but these were not formal regulations.

HIC, high income country; L-MIC, lower middle-income country; U-MIC, upper middle-income country.

In the USA and the United Arab Emirates (UAE), regulation is in part decentralised to the subnational level. For the USA, we purposively selected three focus states based on availability of documents (California, Florida and Nevada). In UAE, we included Dubai which was the only state with specific e-pharmacy regulation. The inclusion of subnational areas was intended to provide some examples of regulation at this level, rather than being a comprehensive overview across all states.

### Search strategy and selection criteria

For each country, we searched official government and regulatory websites for regulations and legal documents, without any restrictions to document type, language or date of issue. We also executed searches on Google and Google Scholar and reviewed the reference lists and cross-referenced documents in included texts to identify additional relevant documents. The full search strategy is reported in online supplemental table 2.

We consulted key informants from each selected country with expertise in pharmacy practice, healthcare services and pharmacy law to ensure that we identified all relevant documents and to ensure correct interpretation of their content. Key informants were purposively selected based on their expertise and professional roles in pharmacy policy, regulation or practice in the selected countries. They were identified through professional networks and snowball sampling and included individuals from regulatory authorities, ministries of health, universities, professional bodies and e-pharmacies. A full list of key informants and their affiliations is provided in the Acknowledgements.

We included all documents explicitly referencing e-pharmacies or synonymous terms (hereafter referred to as ‘e-pharmacy-specific regulations’). We included the latest available (as of 31 January 2025), currently active official regulatory documents (legislation, directives, policies and government-issued mandatory and voluntary guidelines or standards) at country or regional level. Consumer advisories, draft or proposed bills yet to be enforced and consultative documents authored by expert groups leading to regulatory development were not included. Additionally, where an e-pharmacy-specific regulatory requirement was absent, we reviewed general pharmacy regulations to identify applicable requirements even when not explicitly stated. Broader e-commerce, information technology or data privacy regulations not mentioning e-pharmacy were excluded, while acknowledging that these may indirectly apply.

### Data extraction and analysis

We developed a data extraction form based on existing reviews to capture the information provided in each document.^3,6,13–17,19^ A pilot review of four regulatory documents was conducted to test the document inclusion criteria and data extraction tool and finalise guidance for the review team (KN, SNP and GS). This process led to minor refinements to the data extraction tool and helped standardise interpretation of the inclusion criteria. Following the pilot, the identified documents were screened for selection independently and in duplicate by at least two reviewers (KN, SNP and GS). From each included document, we collected information on the title, issuing authority, year of issue, country, purview and subject of legislation and specific text relevant to e-pharmacy. Data from each document were extracted by one reviewer and verified for accuracy by a second reviewer. Non-English texts were translated using Google Translate (interpretation verified by key informants). Any disagreements in screening and data extraction were resolved through discussion between the two reviewers, and if they could not reach consensus, other team members were consulted (CG and BP). Study data were collected and managed using REDCap electronic data capture tool.^22^ Extracted text was analysed in NVivo V.14.0.

We identified regulatory requirements and characterised them into relevant domains. Based on iterative discussions within the research team, we identified 15 ‘basic regulatory requirements’ from the list of requirements, reflecting the most fundamental provisions for any e-pharmacy-specific regulatory instrument. These comprised questions on e-pharmacy licensure, definitions, publicly accessible lists of licensed e-pharmacies, pharmacist requirements, whether online sale of POMs is permitted, prescription requirements, display of pharmacy and pharmacist details, data privacy requirements, recordkeeping and audits; and penalties. These 15 requirements were used as a pragmatic checklist to assess the comprehensiveness of basic regulatory requirements in each country. Countries were scored 1 if their regulations covered a requirement and 0 if not. For countries where sale of POMs was prohibited, prescription requirements were automatically scored as 1 (covered) as they would not be required in this context. The checklist was informed by review of the included regulatory documents and relevant literature.^6,13–17,19,20^

### Patient and public involvement

Patients and members of the public were not directly involved in the design, conduct or reporting of this review, as it focused on analysis of publicly available regulatory documents. However, to strengthen the validity and contextual accuracy of our findings, we consulted key informants in each country to verify the comprehensiveness of the list of identified documents and interpretation of regulatory provisions, and the findings presented incorporate any relevant feedback.

## Results

### Characteristics of regulatory documents identified

We identified 134 documents, of which 41 were included in the review: 32 national, 6 subnational/state-level and 3 supranational (covering EU member states). All but three documents focused on pharmacists or pharmacy providers; the rest addressed clinicians prescribing online. The oldest document was from the USA, that is, Ryan Haight Online Pharmacy Consumer Protection Act (2008), while the latest documents were from the UK and EU (both 2025) (online supplemental table 3).

A definition for e-pharmacy or a related term, such as ‘electronic pharmacy’, ‘online pharmacy’, ‘internet pharmacy’, ‘online sale’ or ‘supply at a distance’, was provided in nine national-level regulations (USA, Brazil, UK, Ghana, South Africa, Kenya, Singapore, Indonesia, UAE), and three subnational regulations (California, Florida and Nevada in the USA). Regulatory definitions of e-pharmacy varied across countries, with the majority emphasising the technological mode of service delivery (Ghana, Singapore, Brazil, South Africa, UK). Regulation from the USA and Nevada framed e-pharmacy through legal restrictions, particularly regarding controlled substances (online supplemental table 4). E-pharmacy was explicitly mentioned but without a definition in the national regulations of Ireland, Germany and Japan, and subnational regulations in Dubai.

### E-pharmacy regulatory requirements by domain

We identified 43 requirements addressed by e-pharmacy regulations and categorised these into 10 key domains: (1) establishment/licensure; (2) accreditation; (3) sale of products; (4) website content; (5) data privacy and security; (6) quality of services and products; (7) advertising; (8) operational oversight and compliance; (9) penalties; and (10) technological and other developments. Domains (1) to (9) were key themes emerging from the documents, while domain (10) was added to capture evolving technological considerations likely to grow in importance.^23,24^ For the purpose of this review, ‘establishment/licensure’ refers to mandatory regulatory approval to operate an e-pharmacy; ‘accreditation’ refers to additional recognition or approval beyond licensure; and certification refers to specific technical assurances, such as display of website security certificates under domain (4).

**Figure 1:**
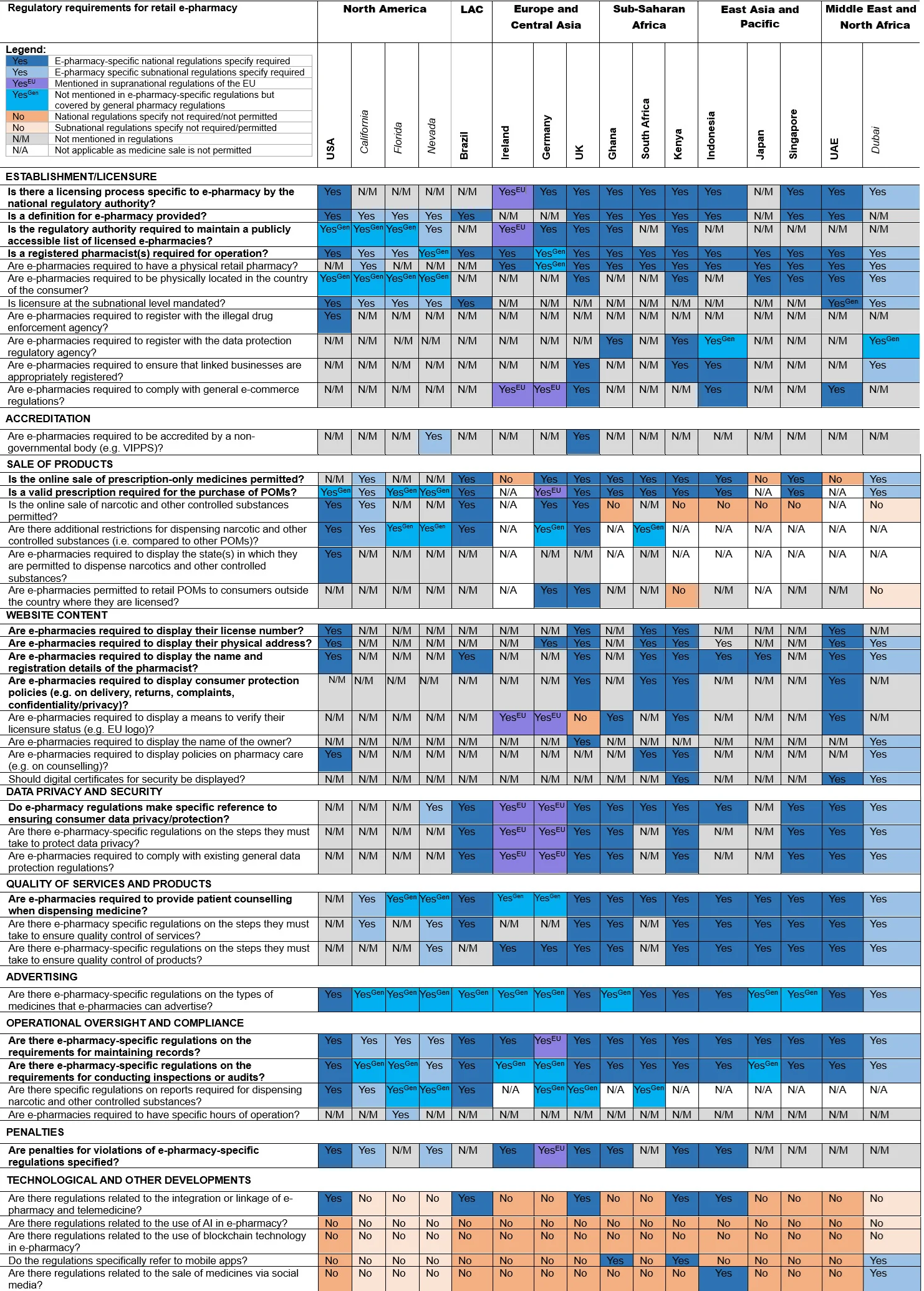
Requirements in e-pharmacy regulations at national, subnational and supranational levels (15 categorised as basic regulatory requirements shown in bold). AI, artificial intelligence; EU, European Union; LAC, Latin America and the Caribbean; POM, prescription-only medicines; UAE, United Arab Emirates; UK, United Kingdom; USA, United States of Ameria; VIPPS, Verified Internet Pharmacy Practice Sites.

Figure 1 summarises the e-pharmacy regulatory requirements, categorised into the 10 regulatory domains.

### Establishment and licensure

Nine national-level documents (USA, Germany, UK, Ghana, South Africa, Kenya, Indonesia, Singapore, UAE) mentioned specific licensing processes for e-pharmacies. At subnational level, the regulations of Dubai mentioned a similar process. The supranational EU regulations mentioned requirements for licensure that member state regulatory authorities were responsible for implementing; of the two EU countries included in our analysis, Germany’s regulations mentioned this requirement, while Ireland’s had a mandate for all retailers to be included on the ‘Internet supply list’ (registry of e-pharmacists maintained by the regulator, Pharmaceutical Society of Ireland). Regulations in Germany, UK, Ghana and Kenya required that a list of licensed e-pharmacies be publicly accessible. EU regulations also required each member state to set up a website, displaying a list of all licensed e-pharmacies. Ghana mandated use of a single National Electronic Pharmacy Platform (NEPP), overseen by the Pharmacy Council for all transactions and monitoring. While not mandated in Indonesia, a publicly accessible list of licensed e-pharmacies or ‘Pharmaceutical Electronic System Operators’ (PSEF) is maintained by the Ministry of Health (https://psef.kemkes.go.id/).

Across all countries included, e-pharmacies were required to operate under the supervision of a licensed pharmacist, as specified either within e-pharmacy regulations or within broader pharmacy legislation (as in Germany and Nevada). Most countries also required any business operating an e-pharmacy to be linked to a physical (brick-and-mortar) retail pharmacy, as explicitly stated in national-level regulations in nine countries (Ghana, South Africa, Kenya, Ireland, UK, Indonesia, Japan, Singapore, UAE). While the national-level documents of the USA did not mention the requirement for a brick-and-mortar pharmacy, the state of California did require this.

Among countries where pharmacy regulation is decentralised, national documents of the USA mandated the licensing of e-pharmacies at the subnational level, and this was also mentioned at the state level in Nevada and California (but not Florida). Dubai mentioned this at the subnational level while UAE’s general pharmacy documents mentioned this at the national level. Brazil mentioned that pharmacists must be licensed with the relevant regional pharmacy council, but there was no requirement for subnational licensing of the e-pharmacy business.

Four countries required authorisations additional to licensing of the e-pharmacy itself. The USA required e-pharmacies to register with the Drug Enforcement Agency (DEA), while Ghana and Kenya required registration with their data protection agencies. The UK required the appropriate registration of all ‘linked businesses’ with the relevant governing bodies (eg, Care Quality Commission, Regulation and Quality Improvement Authority); Dubai required that details of all third parties providing services be approved by the health authority.

### Accreditation

Accreditation requirements were uncommon and appeared jurisdiction-specific. Only one national-level document (UK) mentioned voluntary accreditation, with the General Pharmaceutical Council (GPhC). In the USA, Nevada’s subnational regulations mandated accreditation through the Verified Internet Pharmacy Practice Sites (VIPPS) programme of the NABP; this was not required in California or Florida.

### Sale of products

We identified three broad regulatory approaches to online sale of medicines: jurisdictions that permitted online sale of all POMs, those that permitted online sale of POMs but prohibited narcotics and other controlled medicines, and those that restricted online sale to over-the-counter medicines only.

Online sale of POMs was explicitly permitted in eight national-level documents (Brazil, Germany, UK, Ghana, South Africa, Kenya, Indonesia, Singapore) and in subnational level documents of California and Dubai. In contrast, UAE and Japan prohibited this. The EU allowed member states to determine, at their discretion, whether to permit or restrict the sale of POMs. Germany permitted it; Ireland restricted online sales to over-the-counter medicines. USA’s national and state-level regulations (Florida and Nevada) did not explicitly address online POM sales, though general pharmacy rules appeared to permit them.

In all countries and states in our sample, POMs could only be sold online with a valid prescription. For Germany, this requirement was covered in the EU regulations, which also allow e-pharmacies to dispense e-prescriptions from any member state.

Of those countries allowing online POM sales, four (Ghana, Kenya, Indonesia, Singapore) prohibited online sale of narcotics and other controlled medicines, as did Dubai. Contrastingly, regulations in USA, Brazil, UK and Germany specifically allowed this, though with stricter requirements than for other POMs. The USA also required e-pharmacies to display the states in which they were permitted to dispense controlled substances. South Africa’s regulations made no mention of controlled substances, implying that their sale may be allowed or not explicitly prohibited.

Germany and the UK allowed online POM sales to consumers across borders within the European Economic Area, subject to the laws of the destination country. Dubai regulations prohibited dispensing prescriptions issued by prescribers outside the UAE. Similarly, Kenya only allowed the prescriptions from authorised prescribers in Kenya. No other regulations specified whether sales outside the country of licensure were permitted.

### Website content

Five countries (USA, UK, South Africa, Kenya, UAE) mandated e-pharmacies to display a licence number on their website. E-pharmacies within the EU were required to display the ‘common logo’ (a white cross on a green background with the national flag), linked to a register of licensed online retailers for that country. Outside the EU, the UAE and Ghana also required e-pharmacies to display a similar logo for verification: UAE used the logo of the licensing ministry, while Ghana only mentioned that the Pharmacy Council should provide a logo. Kenya mandated the display of the ‘health safety code’ issued by the regulator for similar verification. Since Brexit in 2021, UK e-pharmacies could no longer display the EU logo, and a replacement system was yet to be established.

Other commonly specified website content included: (1) physical address (n=6; USA, Germany, UK, South Africa, Kenya, UAE); (2) pharmacist details (n=8; USA, Brazil, UK, South Africa, Kenya, Indonesia, Japan, UAE); (3) pharmacy care policies, including counselling (n=4; USA, South Africa, Kenya, Dubai); and (4) consumer protection policies, on delivery, returns, concerns and privacy (n=4; UK, UAE, Kenya, South Africa).

UK and Dubai regulations also required display of the business owner’s name. Kenya, UAE and Dubai also required display of extended-validation (EV-SSL) security certificates, verifying the website’s legal, physical, and operational identity.

### Data privacy and security

Data privacy requirements were present in most countries, but the level of detail varied substantially. Eight countries (Brazil, UK, Ghana, South Africa, Kenya, Indonesia, Singapore, UAE) required e-pharmacies to ensure consumer data protection; all of these specified detailed data privacy safeguards except South Africa and Indonesia. Comparable requirements were outlined at the supranational (EU) and subnational (Nevada and Dubai) levels. Specific privacy measures included storage and transfer of patient data, secure audio-visual communications, safeguarding against cyberattacks, and payment security.

Notably, Singapore required a ‘closed-loop system’ to transfer e-prescriptions directly and securely from the prescriber to the pharmacist, emphasising traceability and the ability to generate an audit trail. For licensure, e-pharmacies in Kenya were required to submit a certificate of registration as a Data Controller from the Office of the Data Protection Commissioner as set forth in the Data Protection Act, 2019.

### Quality of services and products

E-pharmacy-specific service quality control requirements were provided by eight national (Brazil, UK, Ghana, Kenya, Indonesia, Japan, Singapore, UAE) and three subnational (California, Nevada, Dubai) documents. Patient counselling was the most commonly specified service quality requirement, appearing in nine national (Brazil, UK, Ghana, South Africa, Kenya, Indonesia, Japan, Singapore, UAE) and two subnational (California, Dubai) regulations. Other common service quality measures included staff training, use of audiovisual tools for provider–patient communication, provision of patient information, special attention to underserved or vulnerable groups, error minimisation and consumer feedback systems.

Product quality control requirements were provided by nine national (Ireland, Germany, UK, Ghana, Kenya, Indonesia, Japan, Singapore, UAE) and two subnational (Nevada, Dubai) documents. These included temperature control (eg, cold chain), proper packaging and storage and protection from moisture, light, contamination and pests. Ghana specifically mentioned measures to enhance traceability and reduce the risk of ‘counterfeit’, expired and/or unauthorised medicines, through recording each transaction with medicine details (eg, batch number, expiry dates). In the EU, the prevention of falsified medicine distribution was also supported by the Falsified Medicines Directive, which mandates a unique two-dimensional barcode identifier for authentication and an anti-tampering device. These enable end-to-end verification across the supply chain to detect falsified medicines.

### Advertising

Across countries, advertising restrictions on POM promotion were common where online sale of such medicines was otherwise permitted. Six countries (USA, UK, South Africa, Kenya, Indonesia, UAE) and Dubai specified which medicines could be advertised online, while others relied on general regulations. Kenya, South Africa, UAE and the UK explicitly banned advertising of POMs. Indonesia prohibited all medicine advertising except for infant formula and processed food for medical purposes. The USA barred advertisements for controlled substances but allowed advertising of other POMs.

### Operational oversight and compliance

Operational oversight requirements focused mainly on recordkeeping, auditability and inspection. All countries included requirements related to recordkeeping. Among countries allowing online sale of narcotics and controlled medicines, only the USA and Brazil mandated the maintenance of specific reports on their sales. Nine national (USA, Brazil, South Africa, Ghana, Kenya, UK, Indonesia, Singapore, UAE) and two subnational (Nevada, Dubai) regulations specified inspections of e-pharmacies by the relevant governing body or audits. Specifications included regular audits of personnel, inventory, service quality, records, systems to receive and dispense prescriptions, information technology equipment and customer feedback. While most required inspections by regulators, Singapore and Dubai also mentioned regular self-audits. Kenya and Singapore required a verifiable ‘audit trail’. Dubai mentioned monthly self-audits; Ghana mentioned inspections by the Pharmacy Council at least every 6 months.

### Penalties

Penalty provisions were not commonly specified across countries. Four national (USA, Ireland, Ghana, UK), two subnational-level (California, Nevada) and EU regulations specified penalties, which included withdrawal of approval or licence revocation, fines and prison sentences. California imposed a US$150 fine for failure to renew a pharmacy licence, while USA national and Nevada regulations mentioned prison sentences up to 15 years for offences involving controlled substances. The EU left the role of specifying penalties to member states. Kenya’s regulator could suspend or revoke the licence for closed e-pharmacies or those breaching licensure conditions.

### Technological and other developments

Regulation of newer technological developments remained limited. Only five countries (USA, Brazil, Kenya, Indonesia, UK) mentioned regulations related to integrating or linking e-pharmacy with the broader realm of telemedicine. No regulations explicitly mentioned artificial intelligence or blockchain use. Only Ghana, Kenya and Dubai mentioned mobile applications for e-pharmacy. None addressed social media use in online medicine sales, except Dubai, which only mentioned compliance with all federal and local laws related to health advertisements and social media.

### Comprehensiveness of regulatory documents

**Figure 2:**
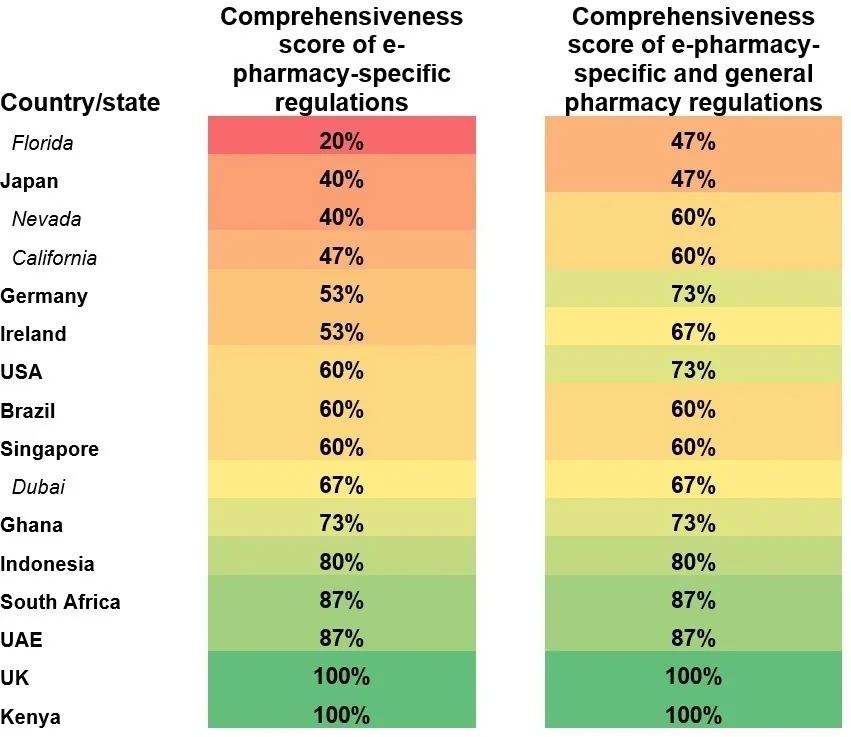
Comprehensiveness scores showing coverage of the 15 basic regulatory requirements for e-pharmacy in (1) e-pharmacy-specific and (2) e-pharmacy-specific and general pharmacy regulatory documents. We identified 15 basic regulatory requirements (marked in bold on figure 1), which indicate the minimum requirements that should be specified in regulations specific to e-pharmacy. A score of 1 was assigned if the regulations covered a requirement and 0 if not. UAE, United Arab Emirates.

We assessed e-pharmacy-specific regulations for 15 basic regulatory requirements (bolded in figure 1) both for e-pharmacy specific regulations as well as general pharmacy regulations where relevant (figure 2). For e-pharmacy specific requirements, the most comprehensive jurisdictions were Kenya and UK, both scoring 100%, that is, providing specific text on all 15 requirements. Three MICs—South Africa (87%), Indonesia (80%), Ghana (73%)—scored higher than all HICs except UK and UAE (87%).

Comprehensiveness scores were the lowest for Florida’s subnational regulations (20%), and Japan’s national regulations (40%). All states in the USA scored below 50%, while EU member states scored slightly above 50%. Including general pharmacy regulations made no difference in nine cases but increased the comprehensiveness scores for the other countries and states, notably increasing the ranking of Germany, the USA and Ireland.

## Discussion

This review found both substantial variation and areas of convergence in e-pharmacy regulatory frameworks across countries. We identified 43 regulatory requirements across 10 domains. While the domains were broadly consistent, regulatory requirements within each domain varied widely—particularly regarding the authorisation to sell prescription or controlled medicines. We also observed considerable regulatory diversity within regions and income groups, suggesting that variation may be shaped by multiple factors including differences in governance, legal architecture, regulatory capacity and the maturity of digital health systems. This diversity was also reflected in the level of government responsible for regulating e-pharmacy, and the role of e-pharmacy-specific regulation vs general pharmacy regulation.

Of the 15 basic requirements that we identified, only the UK and Kenyan regulations met all 15. In general, e-pharmacy-specific regulations in our sample were more comprehensive in MICs than in most HICs. It should be noted that these 15 requirements represent the minimum standards observed in our sample, rather than best practices, such as those specified in the USA VIPPS.^25^ Given the absence of WHO guidance on e-pharmacy regulation, and the lack of specific reference to e-pharmacy in WHO’s Global Benchmarking Tool for regulators,^26^ our 15-item checklist could offer a structured foundation for regulators developing or strengthening e-pharmacy-specific regulatory frameworks. The checklist can help ensure that core provisions are not overlooked. Going forward, it would be beneficial to present the checklist to international groups of regulatory experts for validation.

While this review aimed to provide a comprehensive assessment of e-pharmacy regulations in the 12 countries, several limitations must be acknowledged. First, despite extensive searches and country key informant consultations, reliance on publicly available documents may have led to omissions. Second, there was also a risk of misinterpretation, especially among translated or ambiguous documents, as back translation was not performed. However, we attempted to mitigate this through key informant consultations and verification. Third, for feasibility reasons, we could not include all countries or all states within decentralised systems with enacted e-pharmacy regulation; rather, we purposively sampled to capture regulatory and geographical variation, and the analysis should not be interpreted as globally representative. Fourth, while we reviewed e-pharmacy-specific and general pharmacy regulations where relevant, some requirements marked ‘not mentioned’ in figure 1 may exist in broader e-commerce or data protection regulations. However, it could be argued that to maximise clarity and support compliance, all key regulations should be mentioned or at least cross-referenced within e-pharmacy-specific regulations. Finally, our analysis does not reflect regulatory enforcement or performance; assessing implementation was beyond this review’s scope and warrants future research.

Despite these limitations, the review identifies important areas of regulatory variation, allowing countries to compare approaches and draw lessons when developing or strengthening e-pharmacy frameworks. Countries adopted distinct mechanisms for verifying the legitimacy of e-pharmacies: public registries, common logo and voluntary accreditation. Where supported by sufficient investment and regulatory capacity, such systems could potentially enhance public trust in e-pharmacy, though it remains uncertain whether the general public is familiar with these mechanisms or able to use them effectively. Ghana’s NEPP (https://gnepplatform.com/) goes a step further by requiring all e-pharmacies to be registered on one national platform. However, such administrative centralisation may not be feasible in larger markets, such as the USA, India, or China.

Most countries in our sample, including HICs and LMICs, prohibit standalone e-pharmacies, requiring linkage to brick-and-mortar pharmacies, likely to ensure accountability, facilitate inspections and promote compliance. Importantly, such linkages also eliminate foreign players, restricting the online marketspace to local entities governed by national laws. Our recent analysis of e-pharmacy websites in India and Kenya supports this, showing higher regulatory compliance among local operators than foreign counterparts.^7^ However, such requirements may limit market entry and reduce access gains, particularly in large decentralised systems with areas of low physical pharmacy density or ‘pharmacy deserts’.^2^

The sale of POMs, particularly narcotics and other controlled medicines, demands stringent regulation aligned with national and international rules.^27^ A common approach involves banning online narcotics sales entirely to prevent misuse,^28^ though this was not universal. Variation in these rules may reflect differing regulatory risk appetites and enforcement capacities, as well as differences in how countries balance misuse prevention against timely access for patients with legitimate clinical need. Cross-border enforcement against foreign rogue players remains a significant challenge across all income settings, and it is generally not explicitly addressed in existing frameworks.

Observed regulatory variations raise questions about how different models impact e-pharmacy markets and the safety, quality and equity of care. While stricter regulations may enhance safety, a US study reported that states with less restrictive regulations (eg, removing geographical limits) significantly improved access to medicines, especially among underserved populations.^2^ However, this finding was in the context of the USA’s tightly regulated market. In countries with weaker regulatory oversight, looser regulations could magnify pre-existing risks, including widened access to substandard and falsified medicines.

Some LMICs had already adopted regulations from other countries within this review; for example, Kenya cited the UK’s Medicines and Healthcare products Regulatory Agency guidance and shared overlapping text with South Africa, although their frameworks varied considerably. Supranational regulations, like in the EU, may facilitate regulatory harmonisation and cross-border trade. Similar supranational frameworks may benefit other regions where legal frameworks, regulatory systems and digital systems are sufficiently aligned, particularly in countries that are already part of regulatory harmonisation initiatives. A SALIENT Advisory report noted that Anglophone African countries (Kenya, Ghana, South Africa) lead in e-pharmacy regulatory development, which remained nascent in Francophone Africa.^6^ Mature African regulators could spearhead supranational efforts, possibly under the African Medicines Agency, strengthening regional governance. With robust national surveillance, safe cross-border e-pharmacy sales could bridge access gaps in Africa. Supranational models may also be feasible in the context of the Gulf region, where countries share relatively similar health system structures, ‘somewhat comparable’ national regulations for e-pharmacy and more comparable levels of digital maturity.^17^

National-level regulations in federated countries (eg, USA) complement subnational laws. Decentralised models may allow a division of labour where national authorities address issues such as opioid misuse and foreign rogue players, while state boards manage licensing. However, it may also produce less comprehensive or inconsistent subnational regulation, as observed in Nevada and Florida. This highlights the need for minimum national standards specific to e-pharmacy. Further, without concerted effort to harmonise requirements across states and with national authorities, disparities in medicine quality and pharmacy care may arise, particularly given the borderless nature of e-pharmacies.

Ultimately, the effectiveness of any framework—supranational, national or subnational—depends on enforcement capacity. One way to strengthen enforcement capacity is to integrate e-pharmacy regulation with adjacent regulatory and enforcement systems. In the USA, e-pharmacies selling controlled substances must register with the DEA, the federal agency overseeing controlled substances. As digital health ecosystems evolve, similar integration will be needed between e-pharmacy, telemedicine and other digital health services. In India, most e-pharmacies also offer doctor consultations and home laboratory tests, requiring compliance with medical and digital health regulations.^7^ Data protection is another crucial area for such integration; Kenya and Ghana mandate registration with data protection agencies to safeguard consumers from fraud and privacy breaches. Advanced measures, such as Kenya’s and UAE’s mandatory display of extended validation certificates and Singapore’s ‘closed-loop’ system for secure e-prescription transfer, leverage technology to confer security.

Regulations must evolve with emerging technologies. However, most countries’ requirements did not address the widespread introduction of mobile e-pharmacy apps, medicine sale through social media or use of artificial intelligence and blockchain.^7^ This underlines the need for an agile regulatory system that can respond to rapid technological and market developments. For instance, the UK GPhC recently enhanced its guidelines with new safeguards for weight management medicines following their online misuse.^29^

## Conclusion

Our review found substantial variation in how e-pharmacy regulations addressed key domains, particularly licensure, medicine sale and website authenticity. Gaps were most evident in accreditation, penalties, newer technological aspects and integration with broader digital health systems. The 15-item practical checklist of basic requirements, alongside a broader repository of regulatory options, offers regulators a pragmatic starting point for developing or strengthening existing frameworks. Policy priorities include establishing clear licensing and verification processes, adopting context-specific rules for prescription and controlled medicines that balance access against misuse and integrating e-pharmacy oversight with broader data protection, telemedicine, and digital health systems. Regulatory approaches should also reflect governance context: decentralised models may also require minimum national standards to reduce inconsistency, while countries that are part of regulatory harmonisation initiatives may benefit from supranational models. Where supported by adequate regulatory capacity, adaptive and comprehensive regulatory frameworks can help realise the benefits of e-pharmacy for access to quality pharmacy services while preventing the risks of poor practice.

## Supplementary material

10.1136/bmjph-2025-004309 online supplemental file 1

## Data Availability

All data relevant to the study are included in the article or uploaded as supplementary information.
