# Supplementary material for: Variation and gaps in online pharmacy regulation: a comparative review of regulations across 12 countries
Source: BMJ Public Health. 2026 Sep 18;4(3):e004309. doi: 10.1136/bmjph-2025-004309 (PMC13599940; doi:10.1136/bmjph-2025-004309)
Supplement: Supplementary file 1 [file bmjph-4-3-s001.docx]

**Supplementary file**

**Supplementary Table S1:** Summary of reviews/overviews of e-pharmacy regulations

|  | **REPORTS/GUIDES** | | | | | | | **ACADEMIC LITERATURE** | | | |
| --- | --- | --- | --- | --- | --- | --- | --- | --- | --- | --- | --- |
|  | [**SALIENT**](https://www.salientadvisory.com/2022/11/23/online-pharmacy-in-africa-supporting-pharmaceutical-regulatory-agencies-to-advance-access/) (2023) | [**Baker Mackenzie**](https://www.bakermckenzie.com/-/media/files/insight/topics/healthcare-life-sciences/baker-mckenzie-telemedicine-and-telehealth-legal-f.pdf?sc_lang=en&rev=45433bcd4275493da4df9947c9f87588&hash=22924D77F436913ECF9A844DF2B222FB) (2022) | [**Safir Anand**](https://law.asia/india-healthtech-regulations/#:~:text=Doctors%20must%20follow%20existing%20ethics,on%20its%20movement%20and%20use.) (2022) | [**International Pharmaceutical Federation**](https://www.fip.org/file/5082) (2021) | [**Hock et al. (ISPE)**](https://ispe.org/pharmaceutical-engineering/november-december-2019/regulating-online-pharmacies-medicinal-product-e) (2019) | [**Visa Inc.**](https://bd.visa.com/content/dam/VCOM/global/support-legal/documents/online-pharmacy-guide-for-acquirers-vbs-18-apr-2016.pdf) [**and LegitScript**](https://bd.visa.com/content/dam/VCOM/global/support-legal/documents/online-pharmacy-guide-for-acquirers-vbs-18-apr-2016.pdf) (2016) | [**LegitScript**](https://www.legitscript.com/wp-content/uploads/2016/06/Country_by_Country_-_Internet_Pharmacy_Regulations_-_3-23-15.pdf) (2016) | [**Alfageh et al.**](https://pmc.ncbi.nlm.nih.gov/articles/PMC11321968/) (2024) | [**Rojas-Cortes et al**](https://doi.org/10.26633/RPSP.2023.81)**.** (2023) | [**Miller et al.**](https://gh.bmj.com/content/6/5/e005405) (2021) | [**Sabrina et al.**](https://doi.org/10.3389/fneur.2020.581649) (2021) |
| **Title** | Online Pharmacy in Africa: Landscape of Regulations | Telemedicine and Telehealth Legal Framework Overview | A comparison of healthtech regulations: India | Online pharmacy  operations and  distribution of  medicines:  Global Survey Report | Regulating Online Pharmacies &Medicinal Product e-commerce | Online Pharmacy Guide for Acquirers | Internet Pharmacy Guide: Country Shipping regulations | Overview of online pharmacy regulations in Saudi Arabia and the Gulf cooperation council countries | Regulation of the sale of medical products by  Internet: experience and strategies for Latin America | When technology precedes regulation … | Telemedicine Guidelines in South  East Asia—A Scoping Review |
| **Year** | 2023 | 2022 | 2022 | 2021 | 2019 | 2016 | 2016 | 2024 | 2023 | 2021 | 2021 |
| **Countries** | 1. Benin 2. Burkina Faso 3. Cote D’Ivoire 4. Ethiopia 5. Ghana 6. Guinea 7. Kenya 8. Mali 9. Mauritania 10. Niger 11. Nigeria 12. Rwanda 13. Senegal 14. South Africa 15. Togo 16. India 17. Indonesia 18. Pakistan 19. Singapore | 1. Australia 2. China 3. Indonesia 4. Malaysia 5. Philippines 6. Singapore 7. Taiwan 8. Thailand 9. Vietnam | 1. India 2. China 3. Japan | 1. Africa (14) Eastern Mediterranean (5) 2. Europe (36) 3. Americas (9) 4. Southeast Asia (3) 5. Western Pacific (12) | 1. USA 2. Canada 3. Germany 4. Great Britain 5. Netherlands 6. Australia 7. China 8. Japan 9. South Korea 10. Russia 11. India 12. Singapore 13. Malaysia 14. Indonesia | 1. USA 2. Australia 3. Brazil 4. Canada 5. China 6. France 7. Germany 8. Indonesia 9. Ireland 10. Italy 11. Japan 12. Korea 13. Netherlands 14. Russia 15. Spain 16. Thailand 17. Turkey 18. UK | 1. USA 2. Australia 3. Brazil 4. Canada 5. China 6. France 7. Germany 8. Ireland 9. Italy 10. Japan 11. Korea 12. Netherlands 13. Russia 14. Spain 15. Thailand 16. Turkey 17. UK | 1. Saudi Arabia 2. Bahrain 3. Oman 4. UAE 5. Qatar | 1. Argentina 2. Brazil 3. Colombia 4. Chile | 1. India 2. Kenya 3. Nigeria | 1. Indonesia 2. Malaysia 3. Singapore 4. Thailand 5. Vietnam |
| **Aim** | To assess baseline status of e- pharmacy regulations across African countries  To explore opportunities for advancing regulations. | To address the recent developments and future outlook across telemedicine /telehealth sectors  (more focus on drug distribution and operation of e-pharma) | To explore prevalent intellectual property issues that impact and enable the country’s competitive healthtech sector. | To provide a global snapshot of online pharmacy operations and safety and determine their impact | To provide a global review of current efforts, regulatory status, and way forward for e-pharmacies. | To provide a manual for acquirers who have boarded/considering boarding an e- pharmacy or e-pharmacy referral merchant. | To provide shipping regulations for registrars, registries, and hosting providers. | To provide an insight into the regulations governing e-pharmacy services in the GCC countries. | To present advances in regulation of the sale of medical products  through the Internet in  Latin America  and offer guidance to the NRAs | To examine e-pharmacy  regulatory systems in Kenya, Nigeria, and India | To explore and compare guidelines on telehealth and telemedicine in South  East Asian countries |
| **Focus areas** | - Licensure process - Product types - Provider role - Data privacy - Linkage to other systems | Permissions for:   - Online sales - Delivery - Licensure - E-consults - Recent developments/ future outlook | - Present status of e-pharmacies and telemedicine guidelines - Data protection - Copyright - Brand - Patents - Domain names - Customs registration. | - E-prescription - Online access - Presence of regulations - POMs allowed? - Verification systems - Physical address - Advertisement requirements - Other services - Strengths - Risks - Impact on B&M | Current consumer safety efforts   - USA legal restrictions - FMD directive - Int. law - Accredit. Systems - .pharmacy domain   Approaches of RAs globally   - Online sales permitted? | Legal requirements in major e- pharmacy target markets   - Permitted? - License for importing countries - Drug approval (for sale) in importing countries - Personal imports - Physical consult before online purchase? | Shipping regulations in major e-pharmacy target markets:   - Permitted? - Physical location - Licensure - Import - Consultation mandates - Legitimate resources | - Regulatory bodies - Products sold - Quantities - International sales | - Authorization - Collabs - Surveillance - Health education strategies | - Regulatory bodies - Status of regulations - B&M regulations |  |
| **Methods** | Desk review of e- pharmacy laws, policies, drug/medicine policies, e-health/ cyberhealth strategies and guidelines across the 19 countries.  KI interviews | Overview provided by partners in each country. | Overview provided by legal experts in each country | Survey of respondents from pharmacy organizations.  Results presented descriptively by regions (not by country) | Global review of literature, market reports, and legal documents. | Global guidance based on e-pharmacy market database maintained by LegitScript, in collaboration with Visa Inc. | Global guidance based on e-pharmacy market database maintained by LegitScript | Document review and qualitative interviews with key stakeholders in e-pharmacy | Review of literature and  programs for the control of electronic commerce by agencies. | Drawing on existing literature  KI interviews | Review of academic and gray literature (policy statements, advisories,  blueprints, executive summaries, and circulars) related to telemedicine guidelines. |
| **Key findings** | Most regulations in development stage.  Where e-pharmacy regulations exist, restrictions on eligible products, physical location requirements, and provider roles may impact market growth and represent opportunities for support.  Existing regulations require e- pharmacies to be linked  to retail stores and limit dispensing roles to qualified pharmacists.  Anglophone countries appear to be the most advanced. | Online sale is permitted in all select countries except   - Taiwan: Class B OTC drugs only - Thailand: Telepharmacy notification under development - Vietnam: Unclear; Prescription drugs can only be sold in physical stores but OTC may be sold online. | Only the e-pharmacy developments in India are discussed. | E-prescriptions highest in Europe, and Americas.  Majority of countries reported online operations through brick and mortar pharmacies.  Only 36 countries (49%) reported that e-pharmacy laws existed, of which 26 were from Europe. | Many regulatory authorities lack legislation to properly regulate online pharmacies.  The lack of legislation to mandate private organizations’ cooperation in investigations also impacts enforcement efforts negatively. | Online sale of POMs prohibited in China France, Ireland, Italy, Japan, Russia, Spain, .  Online sale of OTCs also prohibited in South Korea and Turkey.  Regulations are unclear in Indonesia | Online sale of POMs prohibited in China, Ireland, Italy, Japan, Russia, Spain, Thailand, and Turkey. | The existing regulations for online ordering of medications are somewhat comparable between the GCC countries.   - SA, Bahrain, and Oman allow online ordering of both over-the-counter (OTC) and prescription-only medications (POM) from international pharmacies. Controlled medications were prohibited to be ordered online in SA, UAE, and Bahrain | Following  strategic lines are proposed:   - strengthen regulatory and normative framework; - strengthen - supervisory capacity; - collaboration with authorities and other key actors;   communication and awareness among community and health professionals | None of the study countries had yet enacted a regulatory  framework specific to e-pharmacy. | Only Singapore and Indonesia have guidelines on e-prescriptions,  (No narcotics and  psychotropic drugs).  Vietnam permits  prescribing appropriate treatment, but unclear if tele-pharmacy is included.  in Malaysia, online  prescriptions (other than narcotics and psychotropic drugs) are  only permitted as continuation of care. |

**Supplementary Table S2**: Search Strategy

| For each country of interest, we conducted structured searches of official government and regulatory authority websites and national legal or policy databases to identify regulations and legal documents related to e-pharmacy governance. The full list of websites searched, with hyperlinks, is provided below. Within each website, we conducted searches using combinations of specific keywords, including “**online pharmacy**”, “**e-pharmacy regulation**”, “**internet pharmacy**”, “**distance sale**”, “**medicine sale online**”, and “**remote**”, adapting terms where needed to fit the national context. | |
| --- | --- |
| Ghana | [Ghana Pharmacy Council](https://pcghana.org/) |
|  | [Food and Drugs Authority](https://fdaghana.gov.gh/) |
| Kenya | [Pharmacy and Poisons Board](https://web.pharmacyboardkenya.org/) |
|  | [Ministry of Health](https://www.health.go.ke/) |
| South Africa | [South African Pharmacy Council](https://pharmcouncil.co.za/) |
|  | [National Department of Health](https://www.health.gov.za/) |
|  | [South African Health Products Regulatory Authority](https://www.sahpra.org.za/) |
| United Arab Emirates | [Dubai Health Authority, Government of Dubai](https://www.dha.gov.ae/en) |
|  | [United Arab Emirates Ministry of Health and Prevention](https://mohap.gov.ae/en/home) |
| European Union | [The European Parliament](http://europarl.europa.eu/portal/en) |
|  | [The European commission](https://commission.europa.eu/index_en) |
|  | [EUR-Lex](https://eur-lex.europa.eu/browse/summaries.html) |
| Germany | [Federal Ministry of Health](https://www.bundesgesundheitsministerium.de/en/) |
|  | [Federal Institute for Drugs and Medical Devices](https://www.bfarm.de/EN/BfArM/_node.html) |
| Ireland | [Department of Health](https://www.gov.ie/en/department-of-health/) |
|  | [Pharmaceutical Society of Ireland](https://www.psi.ie/) |
| United Kingdom | [Medicines and Healthcare products Regulatory Agency](https://www.gov.uk/government/organisations/medicines-and-healthcare-products-regulatory-agency) |
| Brazil | [Ministry of Health](https://www.gov.br/saude/en) |
|  | [Federal Council of Pharmacy](https://site.cff.org.br/) |
|  | [Brazilian Health Regulatory Agency](https://www.gov.br/anvisa/en) ([Agência Nacional de Vigilância Sanitária](https://www.gov.br/anvisa/en)) |
| United States | [Drug Enforcement Administration](https://www.dea.gov/) |
|  | [Food and Drug Administration](https://www.fda.gov/) |
|  | [Florida Board of Pharmacy](https://floridaspharmacy.gov/) |
|  | [California State Board of Pharmacy](https://www.pharmacy.ca.gov/) |
| Indonesia | [Nevada Board of Pharmacy](https://bop.nv.gov/) |
|  | [The Food and Drug Authority (Badan Pengawas Obat dan Makanan)](https://www.pom.go.id/) |
| Japan | [Ministry of Health](https://kemkes.go.id/eng/home) |
|  | [Ministry of Health, Labor and Welfare](https://www.mhlw.go.jp/www1/english/index.html) |
| Singapore | [Pharmaceutical and Medical Devices Agency](https://www.pmda.go.jp/english/) |
|  | [Health Sciences Authority](https://www.hsa.gov.sg/) |
|  | [Singapore Pharmacy Council](https://www.spc.gov.sg/) |
| No restrictions were applied on document type, language, or date of publication.  To supplement these searches, we performed targeted Google site searches using country-specific keywords (such as “**online pharmacy**”, “**e-pharmacy regulation**”, “**internet pharmacy**” ”, “**distance sale**”, “**medicine sale online**”, and “**remote**”) combined with domain filters (e.g., **site:.gov**, **site:.org**) to identify additional relevant documents from official and regulatory sources.  We also searched Google Scholar to identify supporting regulatory or policy documents and reviewed reference lists of identified materials. Cross-references within national legislation were followed to capture related laws and regulatory provisions.  Finally, key informants from each selected country were consulted to validate the completeness of identified documents and to highlight any additional relevant regulations not captured through the search process. | |

**Supplementary Table S3**: E-pharmacy-specific regulatory documents identified in each country as of January 2025

| **World Bank Region** | **Country** | | **Year** | **Title of the document** | **Document type** | **Issuing Authority** | **Level of enforcement** | **Subject(s)** |
| --- | --- | --- | --- | --- | --- | --- | --- | --- |
| Sub-Saharan Africa | Ghana | | 2021 | Electronic pharmacy policy | Regulation/Legislation | Ghana Pharmacy Council | National | - Pharmacies - Providers - Users |
|  | Kenya | | 2024 | Guidelines for Registration and Licensing of Premises | Guidance | Pharmacy and Poisons Board | National | All premises involved in drug manufacture and supply |
|  |  |  | 2023 | Guidelines for Good Distribution Practices for Medical Products and Health Technologies in Kenya | Guidance | Pharmacy and Poisons Board | National | Distributors and pharmacists |
|  |  |  | 2023 | Guidelines For Internet Pharmacy Services in Kenya | Guidance | Pharmacy and Poisons Board | National | - Pharmacies - Pharmacists |
|  | South Africa | | 2017 | Rules published in terms of section 35A of the Pharmacy Act 53 of 1974 | Regulation/Legislation | South African Pharmacy Council | National | Pharmacies |
|  |  |  | 2010 | Good pharmacy practice in South Africa | Standards | South African Pharmacy Council | National | - Pharmacists |
| Middle East and north Africa | United Arab Emirates | | 2021 | Standards for telehealth services | Standards | Dubai Health Authority, Government of Dubai | Subnational | - Pharmacies - Pharmacists |
|  |  |  | 2021 | Pharmacy guidelines | Guidance | Dubai Health Authority, Government of Dubai | Subnational | - Pharmacists - Pharmacies |
|  |  |  | 2021 | Telehealth Policy | Regulation/Legislation | Dubai Health Authority, Government of Dubai | Subnational | - Providers |
|  |  |  | 2017 | E-Pharmacy Services Guide in UAE | Regulation/Legislation | United Arab Emirates Ministry of Health and Prevention | National | - Pharmacists |
| Europe and Central Asia | European Union | | 2025 | Regulation of the European Parliament and of The  Council on the European Health Data Space | Regulation/Legislation | The European Parliament and The Council of the European Union | Supranational | - Pharmacists - Providers - Distributors |
|  |  |  | 2014 | Commission implementing regulation (EU) no 699/2014 | Regulation/Legislation | The European commission | Supranational | Pharmacies |
|  |  |  | 2011 | Directive 2011/62/EU of the European parliament and of the council | Regulation/Legislation | The European Parliament and The Council of the European Union | Supranational | - Pharmacists - Wholesalers - Manufacturers |
|  | Germany | | 2021 | Medicinal products act | Regulation/Legislation | Federal Ministry of Justice | National | - Pharmacists - Pharmacies |
|  | Ireland | | 2015 | S. I. No. 86/2015. European Union (amendment of the Pharmacy Act 2007) regulations | Regulation/Legislation | Minister for Health | National | - Over-the-counter suppliers |
|  |  |  | 2015 | S.I. No. 87 of 2015 Medicinal Products (prescription and control of supply) (amendment) Regulations | Regulation/Legislation | Minister for Health | National | - Pharmacies - Distributors |
|  | United Kingdom | | 2025 | Guidance for registered pharmacies providing pharmacy services at a distance, including on the internet | Guidance | General Pharmaceutical Council | National | - Pharmacists |
|  |  |  | 2021 | Medicines and Medical Devices Act | Regulation/Legislation | Medicines and Healthcare products Regulatory Agency | National | - Distributors |
|  |  |  | 2017 | MHRA guidance. Selling human medicines online (distance selling) to the public | Guidance | Medicines and Healthcare products Regulatory Agency | National | - Pharmacist - Pharmacy |
|  |  |  | 2013 | The National Health Service (Pharmaceutical and Local Pharmaceutical Services) Regulations | Regulation/Legislation | The Secretary of State | National | - Pharmacists - Pharmacies |
|  |  |  | 2013 | The Human Medicines (Amendment) Regulation 2013 | Regulation/Legislation | The Secretary of State and the Minister for Health, Social Services and Public Safety | National | - Pharmacists - Pharmacies |
| Latin America and the Caribbean | Brazil | | 2023 | Resolution no. 812 | Regulation/Legislation | Ministry of Health/National Health Surveillance Agency/Collegiate Board | National | - Pharmacists - Providers - All premises involved in drug manufacture and supply |
|  |  |  | 2022 | Resolution no. 727 | Regulation/Legislation | Federal Council of Pharmacy | National | - Pharmacists - Providers |
| North America | United States | | 2024 | Laws and Rules Booklet | Regulation/Legislation | Florida Board of Pharmacy | Subnational | - Pharmacies - Pharmacists |
|  |  |  | 2023 | Additional requirements relating to online pharmacies and telemedicine | Regulation/Legislation | Drug Enforcement Administration | National | - Pharmacists   Providers |
|  |  |  | 2023 | Nevada Admin Code | Regulation/Legislation | Department of Administration | Subnational | - Pharmacists |
|  |  |  | 2023 | Lawbook for Pharmacy | Regulation/Legislation | The Department of Consumer Affairs California State Board of Pharmacy | Subnational | - Pharmacists |
|  |  |  | 2016 | USC 21 Section 829 on prescriptions | Regulation/Legislation | Drug Enforcement Administration | National | - Providers |
|  |  |  | 2009 | 21 CFR Part 1304 | Regulation/Legislation | Drug Enforcement Administration | National | - Pharmacists - Providers |
|  |  |  | 2008 | Ryan Haight Online Pharmacy Consumer Protection Act | Regulation/Legislation | Drug Enforcement Administration | National | - Pharmacists - Providers |
| East Asia and Pacific | Indonesia | | 2024 | FDA Number 14 of 2024 concerning the Supervision of Drugs and Food Distributed Online. | Regulation/ Legislation | The Food and Drug Authority (BPOM) | National | - Providers - Pharmacists |
|  |  |  | 2021 | MOH Regulation Number 14 of 2021 concerning the Standards for Business Activities and Products in the Implementation of Health-Based Risk Business Licensing.  Appendix D.42. Standards for Electronic Pharmacy System Organizers/PSEF | Regulation/ Legislation | Ministry of Health | National | - Providers - Pharmacists |
|  |  |  | 2021 | Guidelines for Health Services Through Telemedicine During the Corona Virus Disease 2019 Pandemic | Guidance | Ministry of Health | National | - Providers |
|  | Japan | | 2022 | Regarding the Delivery of Dispensed Medications from Pharmacies | Regulation/Legislation | Ministry of Health, Labor and Welfare, Pharmaceutical Safety and Environmental Health Bureau | National | - Pharmacists |
|  |  |  | 2019 | Partial revision of the Enforcement Regulations of the Act on Ensuring Quality, Efficacy, and Safety of Pharmaceuticals, Medical Devices, etc | Regulation/Legislation | Ministry of Health, Labor and Welfare, Pharmaceutical Safety and Environmental Health Bureau | National | Pharmacists |
|  |  |  | 2014 | Online sales of OTC - Act to partially amend the Pharmaceutical Affairs Act and the Pharmacists Act | Regulation/Legislation | Ministry of Health, Labor and Welfare, Pharmaceutical and Food Safety Bureau | National | - Pharmacists - Retailers |
|  |  |  | 2013 | Act on Partial Revision of the Pharmaceutical Affairs Act and the Pharmacist Act | Regulation/Legislation | General Affairs Division, Pharmaceutical and Food Safety Bureau | National | - Pharmacists - Pharmaceutical products |
|  | Singapore | | 2020 | Guidance notes on supply of registered therapeutic products through e-pharmacy | Regulation/Legislation | Health Sciences Authority | National | - Pharmacists   Pharmacies |
|  |  |  | 2016 | Health products act (chapter 122d) - health products (licensing of retail pharmacies) regulations 2016 | Regulation/Legislation | Health Sciences Authority | National | - Pharmacists |
|  |  |  | 2015 | Singapore pharmacy council code of ethics | Code of Conduct | Singapore Pharmacy Council | National | Pharmacists |
|  |  |  | 2009 | Guidelines for Telepharmacy | Regulation/Legislation | Pharmaceutical Society of Singapore | National | - Pharmacists - Pharmacies - Retailers |

**Supplementary Table S4**: Definition of e-pharmacy and its synonymous terms as per the regulatory documents

| **Country/State** | **Terminology(s) used in the document** | **Definition provided** |
| --- | --- | --- |
| **USA** | Online pharmacy | *The term ‘online pharmacy’—*  *‘‘(A) means a person, entity, or Internet site, whether in the United States or abroad, that knowingly or intentionally delivers, distributes, or dispenses, or offers or attempts to deliver, distribute, or dispense, a controlled substance by means of the Internet; and*  *‘‘(B) does not include—‘‘(i) manufacturers or distributors registered under subsection (a), (b), (d), or (e) of section 303 who do not dispense controlled substances to an unregistered individual or entity; ‘‘(ii) nonpharmacy practitioners who are registered under section 303(f) and whose activities are authorized by that registration; ‘‘(iii) any hospital or other medical facility that is operated by an agency of the United States (including the Armed Forces), provided such hospital or other facility is registered under section 303(f); ‘‘(iv) a health care facility owned or operated by an Indian tribe or tribal organization, only to the extent such facility is carrying out a contract or compact under the Indian Self-Determination and Education Assistance Act; ‘‘(v) any agent or employee of any hospital or facility referred to in clause (iii) or (iv), provided such agent or employee is lawfully acting in the usual course of business or employment, and within the scope of the official duties of such agent or employee, with such hospital or facility, and, with respect to agents or employees of health care facilities specified in clause (iv), only to the extent such individuals are furnishing services pursuant to the contracts or compacts described in such clause; ‘‘(vi) mere advertisements that do not attempt to facilitate an actual transaction involving a controlled substance;‘(vii) a person, entity, or Internet site that is not in the United States and does not facilitate the delivery, distribution, or dispensing of a controlled substance by means of the Internet to any person in the United States; ‘‘(viii) a pharmacy registered under section 303(f) whose dispensing of controlled substances via the Internet consists solely of—‘‘(I) refilling prescriptions for controlled substances in schedule III, IV, or V, as defined in paragraph (55); or ‘‘(II) filling new prescriptions for controlled substances in schedule III, IV, or V, as defined in paragraph (56); or ‘‘(ix) any other persons for whom the Attorney General and the Secretary have jointly, by regulation, found it to be consistent with effective controls against diversion and otherwise consistent with the public health and safety to exempt from the definition of an ‘online pharmacy’.*  *The reporting requirements of this section apply to every pharmacy that, at any time during a calendar month, holds a modified registration authorizing it to operate as an online pharmacy, regardless of whether the online pharmacy dispenses any controlled substances by means of the Internet during the month.* |
| *California* | Telepharmacy  Remote Dispensing Site Pharmacy | *“Telepharmacy” means a system that is used by a supervising pharmacy for the purpose of monitoring the dispensing of prescription drugs by a remote dispensing site pharmacy and provides for related drug regimen review and patient counseling by an electronic method, including, but not limited to, the use of audio, visual, still image capture, and store and forward technology.*  *“Remote dispensing site pharmacy” means a licensed pharmacy located in this state that is exclusively overseen and operated by a supervising pharmacy and staffed by one or more qualified registered pharmacy technicians, as defined in Section 4132, where pharmaceutical care services, including, but not limited to, the storage and dispensing of prescription drugs and controlled substances, drug regimen review, and patient counseling, are remotely monitored or provided, or both, by a licensed pharmacist through the use of telepharmacy technology.* |
| *Florida* | Internet pharmacy | *An Internet Pharmacy as authorized by Section 465.0197, F.S., is required for any location not otherwise licensed or issued a permit under this chapter, within or outside this state that uses the Internet to communicate with or obtain information from consumers and uses the information to fill or refill prescriptions or to dispense, distribute, or otherwise engage in the practice of pharmacy in this state* |
| *Nevada* | Internet pharmacy  Illegal internet pharmacy | *A licensed pharmacy may practice as an Internet pharmacy only if the pharmacy is certified by the Board pursuant to this section. To be certified by the Board pursuant to this section, a pharmacy must apply to the Board for certification on an application provided by the Board.*  *For the purposes of this section, “pharmacy” includes any person who sells or offers to sell drugs to persons in this State via the Internet.*  *“Internet pharmacy” means a person located within or outside this State who knowingly:*  *(a) Uses or attempts to use the Internet, in whole or in part, to communicate with or obtain information from another person; and*  *(b) Uses or attempts to use such communication or information, in whole or in part, to fill or refill a prescription or otherwise engage in the practice of pharmacy.*  *“Illegal Internet pharmacy” means a person located within or outside this State who is not licensed and certified by the Board pursuant to chapter 639 of NRS to engage in the practice of pharmacy via the Internet and who knowingly:*  *(a) Uses or attempts to use the Internet, in whole or in part, to communicate with or obtain information from another person; and*  *(b) Uses or attempts to use such communication or information, in whole or in part, to:*  *(1) Fill or refill a prescription for a prescription drug for the other person; or*  *(2) Deliver or cause, allow or aid in the delivery of a controlled substance, imitation controlled substance, counterfeit substance or prescription drug to the other person.*  *The term does not include a person who is authorized by the provisions of NRS 453.011 to 453.552, inclusive, to dispense or distribute, unless the person is acting outside of that authorization.* |
| **Brazil** | Telepharmacy | *Telepharmacy is understood as the exercise of Clinical Pharmacy mediated by Information and Communication Technology (ICT), remotely, in real time (synchronous) or asynchronously, for the purposes of promotion, protection, monitoring, recovery of health, disease prevention and other health problems, as well as for solving pharmacotherapy problems, for the rational use of medicines and other health technologies. Only selling medicines and other health products, via platforms or software, is not considered telepharmacy.* |
| **EU** | Sale of products via the internet  Sale at a distance | ***-*** |
| **Ireland** | Supply non-prescription medicinal products at  a distance | ***-*** |
| **Germany** | Sale at a distance | ***-*** |
| **UK** | Registered pharmacies  providing pharmacy  services at a distance,  including on the  internet* | *Examples of the pharmacy services covered by this guidance include:*  *1. a pharmacy service where prescriptions are not handed in by people using pharmacy services but are collected by pharmacy staff, or received by post or electronically*  *2. a delivery service from the registered pharmacy to people in their own home, a care home or a nursing home*  *3. a ‘collection and delivery service’. This includes the use of prescription collection units (PCUs): for example automated prescription collection machines and lockers*  *4. a ‘click and collect’ service*  *5. a mail-order service from a registered pharmacy*  *6. an internet pharmacy service, including one linked to an online prescribing service, whether or not the prescribing service is owned and operated by you or by a third-party business*  *7. a ‘hub and spoke’ pharmacy service – where medicines are prepared, assembled, dispensed and labelled for individuals against prescriptions at a central ‘hub’ registered pharmacy* |
| **Ghana** | Electronic pharmacy (e-pharmacy) | *An ePharmacy is a registered retail pharmacy that engages in the business of distribution or sale, stock, exhibit or offer for sale of drugs through an online portal or any other electronic mode. This refers to a registered online, internet or mail-order pharmacy that operates over the internet and sends the orders to customers through the mail or courier/delivery companies. Electronic pharmacies shall only use the NEPP, either directly or indirectly, to provide electronic pharmacy services.* |
| **South Africa** | Pharmacies operating internet  Sites | *Internet websites which sell medicine (S1-6) directly to the public must be operated by and constitute part of a pharmacy licensed by the National Department of Health (NDOH) and recorded with Council. An Internet site cannot exist/operate independently of a pharmacy.* |
| **Kenya** | Internet pharmacy services | *A registered pharmacy that offers to sell or supply medicines (or other pharmaceutical products) and/or provides other pharmaceutical professional services over the Internet or plans for the supply of such products or provision of such services over the internet* |
|  | E-pharmacy | *E-pharmacy” means business of distribution or sale, stock, exhibit or offer for sale of drugs through web portal or any other electronic mode* |
| **Indonesia** | Online distribution  Pharmaceutical Electronic System Operator  Electronic Pharmaceutical Services  Telepharmacy | *Online Distribution of Drugs and Food is any activity or series of activities related to the distribution and/or delivery of Drugs, Medicinal Materials, Natural Medicines, Quasi Drugs, Health Supplements, Cosmetics, and Processed Food using electronic transaction media in the context of trade (Article 1 Paragraph 12 FDA Reg No. 14 of 2024)*  *Pharmaceutical Electronic System Operator, abbreviated as PSEF, is a legal entity that provides, manages, and/or operates a pharmaceutical electronic system for pharmaceutical service* *facilities*  *Electronic Pharmaceutical Services is any activity or series of activities that are carried out in relation to pharmaceutical services using an Electronic Pharmaceutical System*  *Telepharmacy is pharmaceutical services using telecommunication technology and information systems to patients at a distance* |
| **Japan** | Online sale  Internet sale | ***-*** |
| **Singapore** | Telepharmacy  e-pharmacy | *“Telepharmacy" is defined as the provision of Pharmacy services by a Serving Pharmacy to one or more Remote sites through computer, video and audio link.*  *“e-pharmacy” is a mode of pharmacy service that utilises technology to better serve patients by providing more convenient access to pharmacy services. This new mode of pharmacy service is provided by licensed pharmacy in Singapore using a secured online platform. Prescriptions are transmitted electronically through a closed-loop electronic interface from the qualified practitioner or collaborative prescribing practitioner to the qualified pharmacist. The qualified pharmacist processes the received valid prescriptions through electronic means, and conducts professional counselling remotely, as needed. The products are then delivered directly to the patients, who enjoy the convenience of not having to go down to a physical retail pharmacy outlet to fill their prescriptions.* |
| **UAE** | E-pharmacy | *A licensed pharmacy that sells and provides over-the-counter medicines and drugs, medical products and general products, and provides other services including drug advice.* |
| *Dubai* | Telepharmacy  Electronic pharmacy services  Teledispensing (online pharmacy) | **-** |
| *Terminology used by the General Pharmaceutical Council in the UK. Other regulatory documents uses multiple other terminology but without a definition, including “Online sales of human medicines (distance selling) to the public”, “Supply or offer to supply human medicines by means of the internet”, and “Sale of medicines to the public at a distance” | | |

**Author Reflexivity Statement**

1. *How does this study address local research and policy priorities?*

E-pharmacy markets are rapidly growing globally and present an opportunity to address the challenges of poor access to and affordability of quality medicines. The rapid growth of e-pharmacy markets has largely outpaced the governance regimes that are supposed to regulate them, raising concerns over consumer safety and public health. This study systematically mapped and compared e-pharmacy regulations across 12 countries to provide an evidence base for national and global policymakers seeking to strengthen regulatory systems.

2. *How were local researchers involved in the study design?*

Researchers from all collaborating institutions in India, Kenya and the United Kingdom were involved in the development of the funding application, study protocol, and the design of all data collection tools used. Researchers from the collaborating institution in Belgium were involved during the data interpretation and manuscript writing stages, given their expertise in pharmacy regulation.

3. *How has funding been used to support the local research teams?*

Each collaborating institution in India, Kenya and the United Kingdom, has been allocated independent research budgets to support the salaries of local research team members, and to fund in-country data collection, procure research equipment and software, and support dissemination, including for local research team members to attend and present at regional- and global-level academic conferences. Capacity strengthening activities have included training sessions on topics such as regulatory theory, and quantitative and qualitative data collection and analysis; mentoring of early career research team members by senior collaborators; and supervised experiential learning opportunities to gain exposure to all aspects of the academic research life cycle, such as research project management, protocol development, data collection tool development, data management, data analysis, conference abstract submission and presentation, peer-reviewed journal article development and submission, systematic literature reviews, research network and partnership building, and continuing professional development. The collaborating institution in Belgium was not involved in the funding agreement.

4. *How are research staff who conducted data collection acknowledged?*

India-based researchers led all stages of document retrieval, screening, data extraction, and coding. All staff directly involved in data collection and analysis are recognised as co-authors on this manuscript, in accordance with ICMJE criteria. Key informants from the study countries, who reviewed sections of the manuscript to check for completeness / accuracy of the data for their settings, provided consent to be listed under “acknowledgments”.

5. *How have members of the research partnership been provided with access to study data?*

All extracted data are stored securely in India, where primary reviewers were based. The dataset (extraction sheets and checklist scoring) was shared with collaborators in the United Kingdom, Kenya and Belgium to support joint analysis and writing.

6. *How were data used to develop analytical skills within the partnership?*

Early career researchers in India developed skills in systematic searching, screening, coding, and analysis of documentary data related to regulation. All research team members were provided collaborative opportunities to contribute to the development of peer-reviewed journal manuscripts. When needed, this experiential learning has been supported through specific training and/or mentoring, as described in the response to question 3 above.

7. *How have research partners collaborated in interpreting study data?*

For each research output arising from the collaboration, individual research team members may choose to be involved based on their expertise, availability and interest. As a principle, research team members directly involved in the analysis and interpretation of that data, are recognised as co-authors of that research output.

8. *How were research partners supported to develop writing skills?*

Research team members from all collaborating institutions, particularly early career research team members, have been encouraged and supported by senior colleagues to develop academic writing skills by leading in the development of study protocols, manuscripts for peer-reviewed journal articles, and abstracts for submission to relevant academic conferences. The collaboration also implemented monthly Journal Club sessions to discuss relevant scientific and policy reports, and as a forum for team members to present and get feedback on work-in-progress.

9. *How will research products be shared to address local needs?*

All research products arising from the collaboration, including peer-reviewed journal articles, are being published open-access to ensure a wide readership and maximum access to the findings in both the study countries and globally. The collaboration has also established National Advisory Committees (NACs) in both study countries comprised of key stakeholder representatives. NACs provide ongoing advice and local expertise to the local research teams, ensuring the research addresses local needs, and serve as links between the respective country research teams and the users and other potential beneficiaries of the research.

10. *How is the leadership, contribution and ownership of this work by LMIC researchers recognised within the authorship?*

At the inception stage, the research collaboration adopted authorship principles guided by internationally accepted standards that recognise the inputs of all team members involved (i.e. the ICMJE criteria for authorship) and a commitment to building equitable research partnerships that facilitate understanding, fairness, awareness of each other’s contexts, trust and mutual exchange of expertise and knowledge among LMIC and HIC collaborators. For this specific research output, all research team members who contributed to this research work are named as co-authors, the majority of whom are drawn from LMIC collaborators.

11. *How have early career researchers across the partnership been included within the authorship team?*

The project is committed to the capacity development of early career researchers, including through experiential learning opportunities to contribute to and/or lead the development of peer-reviewed publications. For this specific research output, the first author and three other co-authors are early career researchers.

12. *How has gender balance been addressed within the authorship?*

The research teams at each of the three collaborating institutions are mixed-gendered, and the number of women and men among the senior research team members is balanced, with individuals representing a wide and complementary range of expertise and disciplines. This composition ensures that the authorship of all research outputs is mixed with respect to gender. For this specific research output, half of the twelve co-authors are women.

13. *How has the project contributed to training of LMIC researchers?*

As described in the responses to questions 3 and 11, capacity strengthening among early career researchers, particularly those based at LMIC institutions, was a core objective of the research collaboration. Capacity strengthening activities have included training sessions on topics such as regulatory theory, and quantitative and qualitative data collection and analysis; mentoring of early career researchers by senior collaborators; and supervised experiential learning opportunities to gain exposure to all aspects of the academic research life cycle, such as research project management, protocol development, data collection tool development, data management, data analysis, conference abstract submission and presentation, peer-reviewed journal article development and submission, systematic literature reviews, research network and partnership building, and continuing professional development. The project has supported an early career team member to acquire additional funding for PhD training.

14. *How has the project contributed to improvement in local infrastructure?*

The research collaboration has not contributed directly to any improvements to infrastructure in the study countries.

15. *What safeguarding procedures were used to protect local study researchers and participants?*

Prior to commencing any research activities in the field, the study protocols and procedures were reviewed and approved by the institutional review boards at each of the three collaborating institutions in India, Kenya and the United Kingdom. For this specific research output based on scoping review methodology, local research team members collected only publicly available information online and engaged only key informants in the reviewed countries to verify results, who agreed to be acknowledged. Consequently, risks to researchers (and key informants) were considered minimal.
